# Trace metals contamination in groundwater and implications on human health: comprehensive assessment using hydrogeochemical and geostatistical methods

**DOI:** 10.1007/s10653-020-00637-9

**Published:** 2020-06-29

**Authors:** K. Brindha, Rajib Paul, Julien Walter, Mou Leong Tan, Mahesh Kumar Singh

**Affiliations:** 1 Hydrogeology Group, Institute of Geological Sciences, Freie Universität Berlin, 12249 Berlin, Germany; 2 Department of Chemistry, Tripura University, Suryamaninagar, Tripura 799 022 India; 3 Department of Applied Sciences, Centre d’études sur les ressources minérales (CERM), Risk Resources Water (R2eau) Research Group, Université du Québec à Chicoutimi, Saguenay, QC G7H 2B1 Canada; 4 Geography Section, School of Humanities, Universiti Sains Malaysia, 11800 Penang, Malaysia

**Keywords:** Heavy metals, Iron, Faecal coliforms, PHREEQC, Empirical Bayesian kriging, Factor analysis

## Abstract

Monitoring the groundwater chemical composition and identifying the presence of pollutants is an integral part of any comprehensive groundwater management strategy. The present study was conducted in a part of West Tripura, northeast India, to investigate the presence and sources of trace metals in groundwater and the risk to human health due to direct ingestion of groundwater. Samples were collected from 68 locations twice a year from 2016 to 2018. Mixed Ca–Mg–HCO_3_, Ca–Cl and Ca–Mg–Cl were the main groundwater types. Hydrogeochemical methods showed groundwater mineralization due to (1) carbonate dissolution, (2) silicate weathering, (3) cation exchange processes and (4) anthropogenic sources. Occurrence of faecal coliforms increased in groundwater after monsoons. Nitrate and microbial contamination from wastewater infiltration were apparent. Iron, manganese, lead, cadmium and arsenic were above the drinking water limits prescribed by the Bureau of Indian Standards. Water quality index indicated 1.5% had poor, 8.7% had marginal, 16.2% had fair, 66.2% had good and 7.4% had excellent water quality. Correlation and principal component analysis reiterated the sources of major ions and trace metals identified from hydrogeochemical methods. Human exposure assessment suggests health risk due to high iron in groundwater. The presence of unsafe levels of trace metals in groundwater requires proper treatment measures before domestic use.

**Electronic supplementary material:**

The online version of this article (10.1007/s10653-020-00637-9) contains supplementary material, which is available to authorized users.

## Introduction

Trace metals occur naturally in the environment, and their presence in groundwater is generally not desired as many have toxic effects even at low concentrations. This is problematic especially in the urban and rural areas where groundwater serves as a major source for drinking water supply. Arsenic enrichment in groundwater is a widely known global issue affecting millions of people living in several countries. Lead, mercury and cadmium in groundwater have also caused adverse effects on human health and the ecosystem. The World Health Organisation’s (WHO) list of top-ten chemicals of major public concern includes these four trace metals (arsenic, lead, mercury and cadmium) due to their high toxicity, persistence in the environment and bioaccumulative nature (WHO 2019). Hence, there is increasing public health and ecological concern in recent years over contamination of the environment from trace metals. Even though trace metals are found in the earth’s crust, contamination in groundwater could be an outcome of natural and/or anthropogenic sources. The aquifer type, intensity of weathering of minerals from the aquifers, precipitation frequency, quality of the infiltrating water and residence time are the natural factors that control the presence of trace metals in groundwater (Chanpiwat et al. 2014; Ghesquière et al. 2015; Magesh et al. 2017). Anthropogenic sources are due to wastes from various industrial activities (e.g. tanning, electroplating, chemicals and textile manufacturing, mining, smelting, etc.), soil contamination, underground storage tanks, landfills, tailings ponds, urban sewage, contaminated surface water, fertilizers and pesticides used for agriculture, etc. (Boateng et al. 2019; Christensen et al. 2000; Yousaf et al. 2016).

Some of the trace metals are essential for the physiological and biochemical functioning of flora, fauna and humans, while few trace metals induce toxicity even at meagre amounts. Hence, these trace metals are classified as essential (iron, manganese, zinc, copper, etc.) and non-essential elements (lead, cadmium, arsenic, etc.) based on public health perspective. Interaction between high concentrations of trace metals and humans occurs through three major pathways: inhalation, ingestion and dermal absorption. Of these human exposure pathways, ingestion in the form of drinking water and food preparation, and dermal contact through domestic activities result from using contaminated water. Public water supply through well-established infrastructure and intensive treatment to meet the guidelines for drinking water supply are common in developed nations (Brindha and Schneider 2019). However, this is not the case in developing nations (such as in India, Myanmar, Laos) wherein water supply is covered in 94% of the urban areas and 76% of the rural areas (WHO, undated). Population not covered by water supply facilities rely on private bore wells extracting the limited groundwater resource. Hence, monitoring the occurrence of trace metals in groundwater is crucial to evaluate the potential human health risk.

India is a large country with nearly 4% of the world’s renewable water resources but hosts about 18% of the world’s population. It also ranks first as the most groundwater using nation (Rodell et al. 2009; Wada et al. 2010). The freshwater demand is increasing due to population growth and the subsequent need to produce more agricultural products to feed the growing population. The limited water resources are unevenly distributed, and there exists huge spatial and temporal variability in the amount of rainfall. Despite these differences, trace metal contamination is reported in all the climatic regions of India, i.e. semi-arid, tropical wet, and dry and humid subtropical zones (Coyte et al. 2019; Kumar and Singh 2019; Kumar et al. 2019; Kumar et al. 2017; Sharma et al. 2019; Sridharan and Nathan 2018). Deterioration of groundwater quality from trace metals mainly due to iron and arsenic from geogenic sources, and chromium from tanneries are well documented in India (Brindha and Elango 2012; Chakraborti et al. 2017a; Ghosal et al. 2015; Kanagaraj and Elango 2019; Nath et al. 2018; Singh et al. 2018).

Tripura, located in northeast India, is one of the regions with demand for groundwater as a freshwater source to supply the increasing population, agricultural and industrial needs. Groundwater meets 80% of rural, 50% of urban, and 50% of irrigation needs (Debbarman et al. 2013). Tripura is rich in water resources; the net groundwater available annually is 1.97 × 10^9^ m^3^, and the groundwater withdrawn is 0.17 × 10^9^ m^3^/year (CGWB, undated). Published information on the status of groundwater quality in West Tripura is scarce. The available information is restricted to the analysis of the drinking and irrigation water quality (Paul et al. 2016, 2019b; Singh and Kumar 2015) and reporting the presence of trace metals in groundwater (Banerjee et al. 2011; CGWB 2012a, b). Nevertheless, the origin and mechanisms controlling the trace metal concentrations in groundwater are not fully understood. There is a pressing need for a comprehensive assessment of the geochemical characteristics of groundwater in this region with special focus on trace metals. Hence, the objective of this study is to identify the origin and the hydrogeochemical processes that are responsible for the elevated concentration of trace metals in groundwater of West Tripura, India. Public health risk to humans from exposure to these trace metals is also quantified.

## Methodology

### Description of study area

**Fig. 1:**
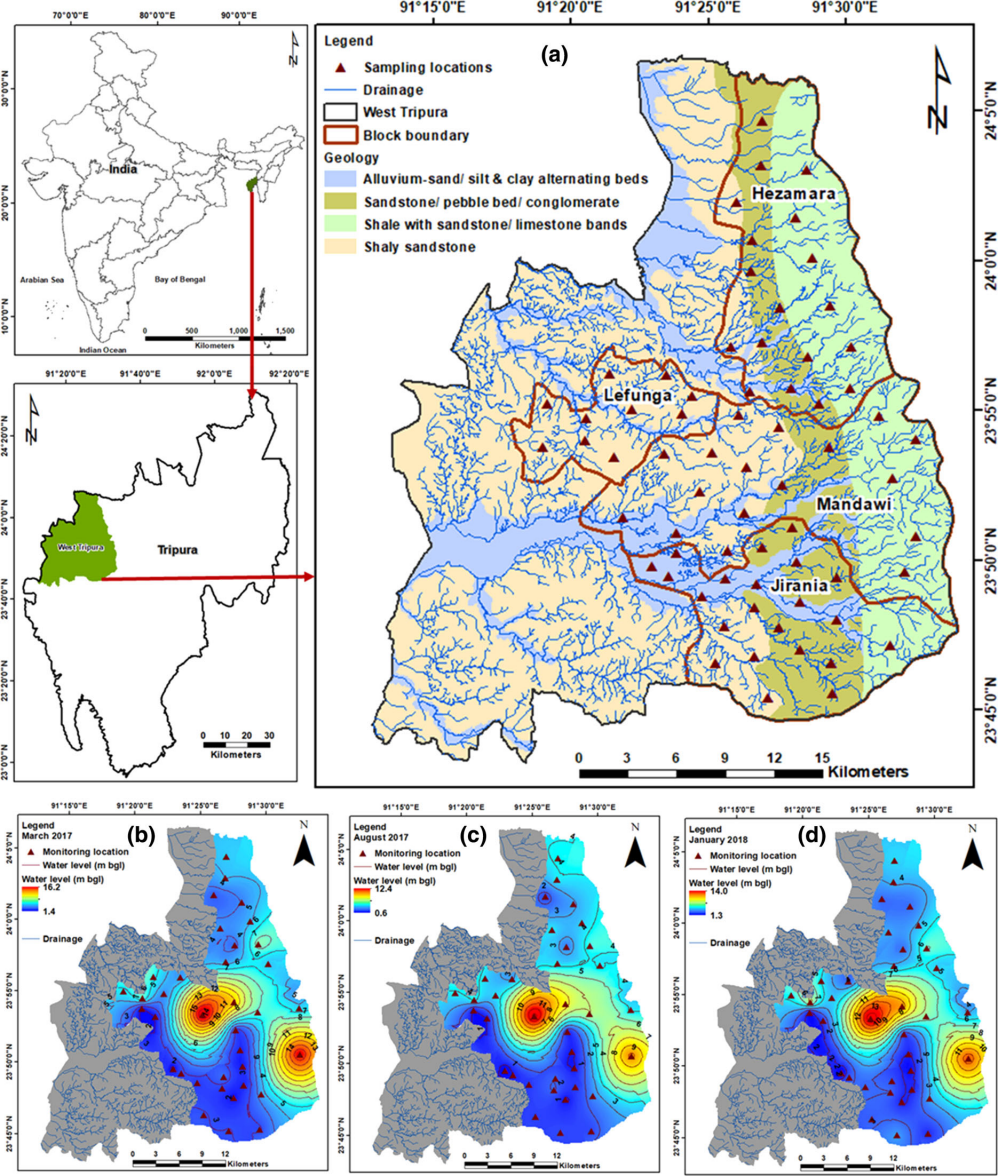
**a** Location of the study area with geology, drainage and monitoring locations. Spatial distribution in groundwater level (m below ground level) in **b** March 2017, **c** August 2017 and **d** January 2018

Study area includes four blocks of West Tripura district (Hezamara, Lefunga, Mandawi and Jirania) and covers an area of ~529 km^2^ (Fig. 1a). Climate is characterized by sub-tropical and temperate zones with high humidity. There are three prominent seasons: summer (March to May), monsoon (June to September) and winter (November to February) (CGWB 2012a, b). Minimum temperature up to 5 °C is experienced in winter, and maximum temperature raises up to 36 °C in summer. Average annual rainfall is about 2000 mm contributed mainly by the Southwest monsoon (IMD 2019). Topography is hilly in the eastern part. There are many undulating plains and wide and long valleys. Drainage patterns commonly noticed are sub-parallel to parallel and dendritic (GSI 2011). Rainfall is the main source of groundwater recharge.

The study area comprises of three main geological formations, namely the Tipam, Dupitila and Bokabil formations. The Tipam formation consists of fine to coarse-grained sandstone that are soft and fragile with occasional bands of siltstones (GSI 2011; Paul et al. 2019a). The sandstone unit contains boulders with outer ferruginous coating and inner calcareous concretions. Dupitila formation overlies the Tipam Group with an angular unconformity and consists of unconsolidated ferruginous sandstone. These sandstones are white to yellowish, and loose with pink and yellow clay bands. Major minerals in these coarse-grained sandstones are grains of quartz, quartzite, feldspar, muscovite and biotite (GSI 2011). Alluvium deposits in the flood plains of recent to sub-recent rivers comprise of unconsolidated sand, silt, clay and decomposed organic matter (GSI 2011; Paul et al. 2019b). The sedimentary rocks in these formations act as potential aquifers due to high porosity (Paul et al. 2019b). In shallow aquifers, groundwater occurs under unconfined conditions.

### Sample collection and analysis

Groundwater samples were collected from 68 locations distributed over four blocks in West Tripura district (Fig. 1a) from 2016 to 2018. Throughout the study, 408 groundwater samples were collected and analysed for 18 parameters. A part of the data (*N* = 45) published earlier was also included in this study for a comprehensive assessment of the groundwater quality in the region (Paul et al. 2016, 2019a). Sufficient care was taken to collect samples from different land use, and that the sampling locations were well-distributed over the study area. Water level was measured in selected Central Ground Water Board (CGWB) monitoring wells and open wells where it was feasible (*N* = 37 during March 2017, August 2017 and January 2018). Other tube wells could not be dismantled, and hence, the water level could not be measured. pH and electrical conductivity (EC) were measured in situ using portable water quality metres (Eutech PCSTestr 35). The digital metres were precalibrated with 4.01, 7 and 10.1 pH solutions and 84 μS/cm and 1413 μS/cm conductivity solutions. Groundwater samples were collected in 500-ml high-density polyethylene bottles which were precleaned by soaking in 2 M HNO_3_and rinsing with deionized water. Bottles were rinsed 3–5 times with the groundwater samples before filling the bottles with the sample.

Major cations (calcium, magnesium, sodium, potassium), major anions (chloride, sulphate, bicarbonate) and minor ions (fluoride, nitrate) were determined through standard procedures (APHA 2012). Samples for trace metal analysis were acidified with HNO_3_ (pH < 2), stored in a cooler and brought to the laboratory for analysis. Iron, copper, cadmium, manganese, arsenic, lead and zinc concentrations in the groundwater samples were analysed using an atomic absorption spectrophotometer (PerkinElmer AAnalyst 700). The percentage error in ionic balance varied up to ± 8%. Detection limits for iron, copper, manganese and zinc are 0.001 mg/l, cadmium and lead are 0.003 mg/l, and arsenic are 0.2 µg/l. Summary of the methods adopted and the detection limits are given in Table S1 (Supplementary material). Standards and blanks were run at regular intervals to ensure accuracy in measurements. Durov diagram to determine the groundwater geochemistry was plotted using Grapher version 17.

### Calculation of saturation indices

Saturation index (SI) helps to evaluate the mineral equilibrium for groundwater samples. This can be useful in predicting the occurrence of reactive minerals and in estimating their reactivity. Geochemical modelling code, PHREEQC (Parkhurst and Appelo 1999), was used to calculate the SI of minerals. This is calculated using the formula, SI = log (IAP/KT), where SI is the saturation index, IAP is the ion activity product of the mineral, and KT is the equilibrium constant. If the saturation index is less than zero, groundwater is undersaturated with the mineral, and if the saturation index is greater than zero, groundwater is supersaturated. In undersaturated condition, the mineral cannot precipitate from solution and has to dissolve to reach equilibrium (Appelo and Postma 2005; Deutsch and Siegel 1997). Minerals in supersaturated state in groundwater tend to precipitate to attain equilibrium. If the saturation index is 0, then the groundwater is in equilibrium with respect to the mineral considered. Due to the uncertainties in calculation of the SI, SI between 0.5 and − 0.5 can be considered as a mineral’s equilibrium zone (Bouzourra et al. 2015; Deutsch and Siegel 1997).

### Statistical analysis

Many statistical methods are available for predicting the spatial concentration of ions. In this study, the empirical Bayesian kriging (EBK), a geostatistical interpolation model that is based on the classic kriging model, was used (Krivoruchko 2012). Recently, many water and soil studies have adopted this method (Boateng et al. 2019; Fabijańczyk et al. 2017; Giustini et al. 2019; Myers and Schultz 2000; Roberts et al. 2014; Samsonova et al. 2017). EBK is different from the classic kriging model in that it does not require manual adjustment of its parameters to acquire precise output. Instead, EBK automates the parameter calculation through sub-setting and simulations. Other key variation between EBK and other kriging models is that it accounts for the errors estimated by the semivariogram. Normally, kriging models use only one semivariogram from the observed data and use this to predict the values in unknown locations. But, EBK accounts for these errors by using several semivariogram models and is carried out in a series of steps (Krivoruchko 2012). Initially a semivariogram is predicted with the collected data. This semivariogram is then used to predict new values for these collected data locations. From these newly predicted data, a new semivariogram model is obtained. These steps are repeated resulting in many semivariograms (Krivoruchko 2011). Weight for the semivariograms is calculated based on Bayes’ rule, and these weights are used to predict standard errors at the unsampled locations. The default and the most flexible K-Bessel model in EBK were used (Krivoruchko 2011). The spatial analysis using EBK was performed using ArcMap 10.4. IBMM SPSS 21 was used to perform multivariate statistical analysis. Factor analysis was carried out with principle component extraction method and varimax normalized rotation. Minimum eigenvalue was set to 1.

### Comprehensive assessment of water quality

Normally, water quality index (WQI) represents the water quality only at one point of time. When samples are collected at different time-periods, usually the WQI is calculated for each time of sampling and an average of this is used to represent the WQI of the location. In the WQI proposed by the Canadian Council of Ministers of the Environment (CCME), the samples collected from one location during multiple sampling campaigns can be combined to provide a comprehensive assessment of the water quality of a location (CCME 2001). Hence, this index guided by three factors: scope (F1), frequency (F2) and amplitude (F3) was adopted in this study.

Scope (F1) represents the percentage of parameters that do not meet the suitable guideline limits relative to the total number of parameters measured during the period of study.

1 $${\text{Scope}} \left( {{\text{F}}1} \right) = \frac{{{\text{Number}}\;{\text{of}}\;{\text{failed}}\;{\text{parameters}}}}{{{\text{Total}}\;{\text{number}}\;{\text{of}}\;{\text{parameters}}}}{ \times }100$$

Frequency (F2) represents the failed tests in a location during the study period. It is calculated as a percentage of individual tests that do not fall within the prescribed limits to the total number of tests.

2 $${\text{Frequency}} \left( {{\text{F}}2} \right) = \frac{{{\text{Number}}\;{\text{of}}\;{\text{failed}}\;{\text{tests}}}}{{{\text{Total}}\;{\text{number}}\;{\text{of}}\;{\text{tests}}}}{ \times }100$$

Amplitude (F3) represents the amount by which the failed parameters do not meet the guidelines, and this is calculated in three steps. Firstly, excursion is calculated as the number of times an individual test is greater or less than the limit. For cases where the test value must not exceed the guideline value (referred to as objective in the equation), Eq. 3 is used and cases where the test value must not be less than the objective, Eq. 4 is used. From the excursion, the normalized sum of excursions (nse) is calculated as a sum of the excursions to the total number of tests conducted for the location during the period of study (Eq. 5). Amplitude is calculated from the nse as in Eq. 6.

3 $${\text{Excursion}}_{i} = \frac{{{\text{Failed}}\;{\text{test}}\;{\text{value}}_{i} }}{{{\text{Objective}}_{j} }} - 1$$

4 $${\text{Excursion}}_{i} = \frac{{{\text{Objective}}_{j} }}{{{\text{Failed}}\;{\text{test}}\;{\text{value}}_{i} }} - 1$$

5 $${\text{Excursion}}_{i} = \frac{{\mathop \sum \nolimits_{i = 1}^{n} {\text{Excursion}}_{i} }}{{{\text{Number}}\;{\text{of}}\;{\text{tests}}}}$$

6 $${\text{Amplitude}}\left( {{\text{F}}3} \right) = \frac{\text{nse}}{{0.01\;{\text{nse}} + 0.01}}$$

Finally, the square root of the squares of the three factors is calculated and divided through 1.732 to normalize the values to range from 0 to 100, where 0 represents poor water quality and 100 represents good water quality (CCME 2001).

7 $${\text{CCMEWQI}} = 100 - \left( {\frac{{\sqrt {{\text{F}}1^{2} + {\text{F}}2^{2} + {\text{F}}3^{2} } }}{1.732}} \right)$$

### Health risk assessment

Human exposure and risk assessment through drinking water pathway (chronic daily intake (CDI_oral_)) (USEPA 2011) and the potential non-carcinogenic risk from trace metals (hazard quotient (HQ)) (USEPA 1989) were calculated using the following equations.

8 $${\text{CDI}}_{\text{oral}} = \frac{{C{ \times }{\text{IR}}{ \times }{\text{EF}}{ \times }{\text{ED}}}}{{{\text{BW}}{ \times }{\text{AT}}}}$$

9 $${\text{HQ}} = \frac{{{\text{CDI}}_{\text{oral}} }}{\text{RfD}}$$

where CDI_oral_ = average daily dose of ingestion of the trace metals (mg/kg-day), C = measured concentration of the trace metal in water (mg/l), IR = average daily water intake (l/day), EF = exposure frequency (days/year), ED = exposure duration (years), BW = average body weight (kg), AT = average life expectancy (days), HQ = hazard quotient and RfD = oral reference dose for a trace metal that an individual can be exposed to in a day over his/her lifetime without experiencing any harmful health effect (mg/kg-day).

## Results and discussion

Maximum depth to water level was 16.3 m, 12.5 m and 14.1 m in March 2017 (summer), August 2017 (monsoon) and January 2018 (winter), respectively (Fig. 1b–d). The mean groundwater level in this area is 4 m bgl (*N* = 111). Range of groundwater level representing premonsoon was 1.8- 16.3 m and postmonsoon was 0.6–14.1 m. Rise in groundwater level was in the range of 0.46 to 5.84 m between March and August 2017 representing impact of rainfall recharge. Decline in groundwater level was in the range from 0.1 to 3.2 m between August 2017 and January 2018 indicating local pumping for various activities. Spatial variation in the average groundwater level shows lower water table near settlements and agricultural areas in the central and eastern part. Descriptive statistics of the composition of groundwater samples is presented in Table 1. Groundwater pH indicates that it is strongly acidic to slightly alkaline in nature. Average EC was 120 µS/cm and total dissolved solids (TDS) were 78 mg/l. Groundwater generally is less mineralized as shown by the EC and TDS values. Overall, groundwater was fresh and soft to moderately hard in nature.

**Table 1:** Detailed statistical summary of parameters measured in groundwater

Parameter	Min	Max	Mean	Acceptable limit (BIS 2012)	Desirable limit (BIS 2012)
pH	4.25	7.95	–	6.5-8.5	No relaxation
EC (µS/cm)	22.80	260.00	120.0	–	–
TDS (mg/l)	14.00	169.00	78.0	500	2000
Ca (mg/l)	3.74	22.47	10.29	75	200
Mg (mg/l)	2.08	13.61	4.97	30	100
Na (mg/l)	0.10	49.71	2.71	–	–
K (mg/l)	0.03	16.69	0.84	–	–
HCO3- (mg/l)	9.48	146.40	40.72	200	600
Cl- (mg/l)	7.68	39.65	17.56	250	1000
SO4-2 (mg/l)	1.35	32.03	7.78	200	400
NO3- (mg/l)	0.13	4.21	0.90	45	No relaxation
F- (mg/l)	0.08	0.58	0.24	1	1.5
Fe (mg/l)	0.05	5.39	1.36	0.3	No relaxation
Mn (mg/l)	0.01	0.62	0.10	0.1	0.3
Pb (mg/l)	BDL*	0.03	0.02	0.01	No relaxation
Cd (mg/l)	BDL	0.01	0.01	0.003	No relaxation
As (mg/l)	BDL	0.05	0.01	0.01	0.05
Cu (mg/l)	BDL	0.04	0.01	0.05	1.5
Zn (mg/l)	0.01	0.10	0.02	5	15
Cr (mg/l)	BDL	0.03	0.01	0.05	No relaxation
TC (MPN/100 ml)	Nil	63.00	10.24	No detection	No relaxation
FC (MPN/100 ml)	Nil	48.00	7.11	No detection	No relaxation

*BDL = below detection limit

### Hydrochemical facies

**Fig. 2:**
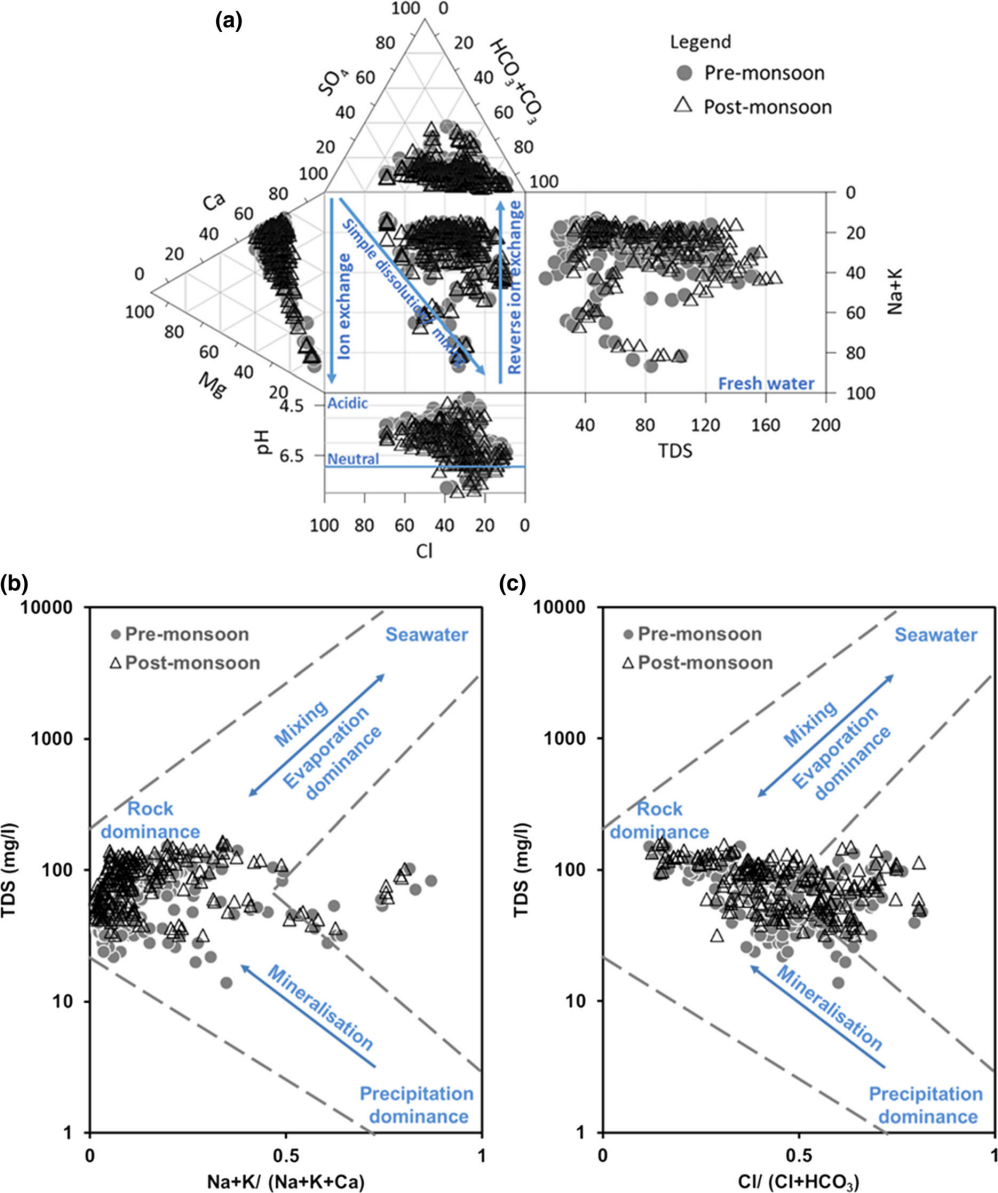
**a** Durov plot showing the hydrogeochemical facies and processes in the study area. **b** Gibbs plot showing the dominant hydrochemical processes

Extended Durov diagram was used to display the relative concentration of the major ions in relation to the TDS and pH. Mixed Ca–Mg–HCO_3_, Ca–Cl and Ca–Mg–Cl were the dominant groundwater types (Fig. 2a). Relatively few samples had Na–Cl or mixed Ca–Na–HCO_3_ water types. From the individual cation and anion trilinear plots, it is evident that calcium is the dominant cation, whereas bicarbonate and chloride were the equally dominant anions. General dominance of cations occurs in the order of Ca^2+^, Mg^2+^, Na^+^, K^+^, and anions were in the order of HCO_3_^−^, Cl^−^, SO_4_^2−^. Bicarbonate (HCO_3_^−^) waters represent freshly recharged water. With longer flow path and residence time, the groundwater tends to change to Cl-type water. Ca-HCO_3_ and Ca–Cl groundwater types were reported in the study area and the adjacent areas in earlier studies (Paul et al., 2019a).

### Groundwater mineralization

Many hydrochemical processes contribute to the water quality changes within the aquifer. To narrow down to the key processes, Gibbs plot using the ion ratios and salinity of groundwater was used (Gibbs 1970). The Gibbs diagram makes it possible to distinguish between water dominated by water–rock interactions (rock dominance), by seawater mixing and evaporation processes, or by freshwater inflow (recharge by rainwater). Water samples were grouped mostly in the rock dominance part of the Gibbs plot (Fig. 2b, c). Evaporation did not play a significant role in governing the hydrochemistry. Few samples relate similar to rainwater and could be attributed to recently recharged water. Lower Na/(Na + K+Ca) ratio specifies the dominance of carbonate minerals, and higher values represent silicate dominance. Gibbs plot was originally developed for surface waters and hence does not provide detailed information on the other processes such as that involving SO_4_ (Marandi and Shand 2018). Similarly, although the change in the HCO_3_ to Cl ratio is captured through Gibbs plot, the changes in the Ca–Mg ratio are not observed. But, this plot could be adopted for groundwaters (Marandi and Shand 2018) and the key governing processes identified through this plot can be used to further refine the hydrogeochemical processes.

#### Weathering and dissolution

**Fig. 3:**
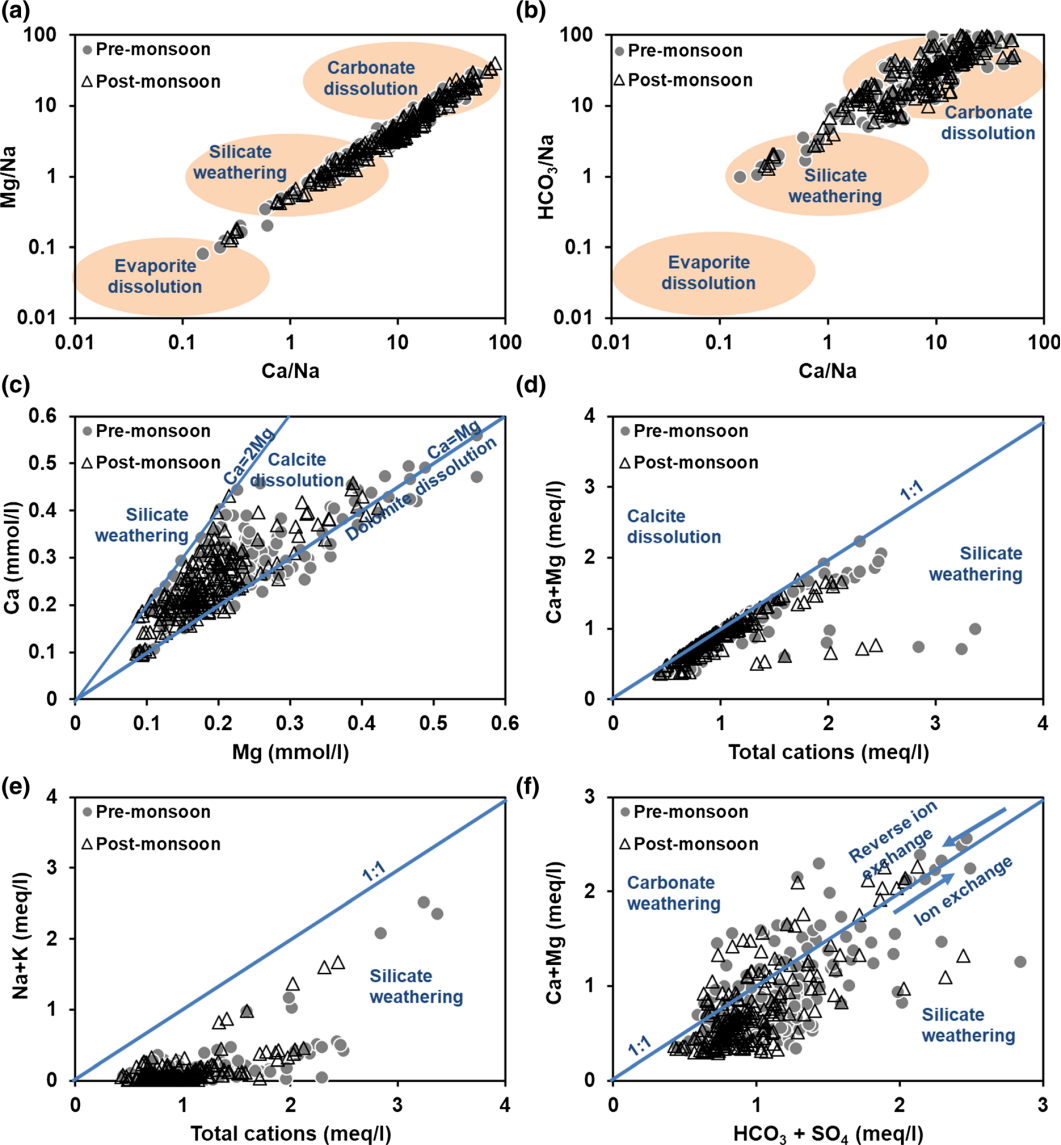
Bivariate plots explaining the geochemical processes

Figure 3a, b plotted with ratios of Ca/Na against Mg/Na and HCO_3_/Na shows the evaporite, silicate and carbonate dissolution processes. Hydrochemistry in this area is governed by silicate weathering and carbonate dissolution. Molar ratios of calcium (mCa) and magnesium (mMg) also confirm these processes (Fig. 3c). In Fig. 3c, mCa = mMg indicate dolomite dissolution, 2 > mCa/Mg > 1 indicate calcite dissolution, and mCa = 2mMg indicate silicate weathering (Ghesquière et al. 2015; Rajmohan and Elango 2004). Values of mCa/mMg ranged from 0.8 to 2.0. With an average molar ratio of 1.3, 97% of the groundwater samples showed the influence of dolomite and calcite dissolution over silicate weathering.

Calcium and magnesium dominance in comparison with Na + K is depicted through the plots of total cations versus Ca + Mg (Fig. 3d) and versus Na + K (Fig. 3e). In Fig. 3d and e, groundwater samples are aligned with the equiline for Ca + Mg and deviate more from the 1:1 equiline in case of Na + K. This reflects an increasing contribution of Ca + Mg with increase in TDS. Several data points also lie below the 1:1 equiline which are likely to be derived from silicate weathering. Figure 3e showing the contribution of Na + K to the total cations by falling below the 1:1 line further confirms that silicate weathering is responsible for Na and K in groundwater (Kanagaraj and Elango 2019; Senthilkumar and Elango 2013).

Bivariate plot of Ca + Mg and HCO_3_ + SO_4_ provides evidence on carbonate and silicate weathering processes. This plot not just explains the dissolution and weathering processes, but also the occurrence of ion exchange and reverse ion exchange. Amount of Ca^2+^and Mg^2+^ gained or lost relative to that provided by the dissolution of Ca^2+^and Mg^2+^-bearing minerals is reflected here. If the data fall on the 1:1 line, they result from carbonate (calcite, dolomite) and sulphate minerals (gypsum, anhydrite) (Masoud et al. 2018). In Fig. 3f, the samples from the study area are not only distributed on the 1:1 equiline but are also placed away from this line. Data points on the Ca + Mg side indicate excess of these ions and are derived from reverse ion exchange. Samples placed on the HCO_3_ + SO_4_ shows direct ion exchange.

**Fig. 4:**
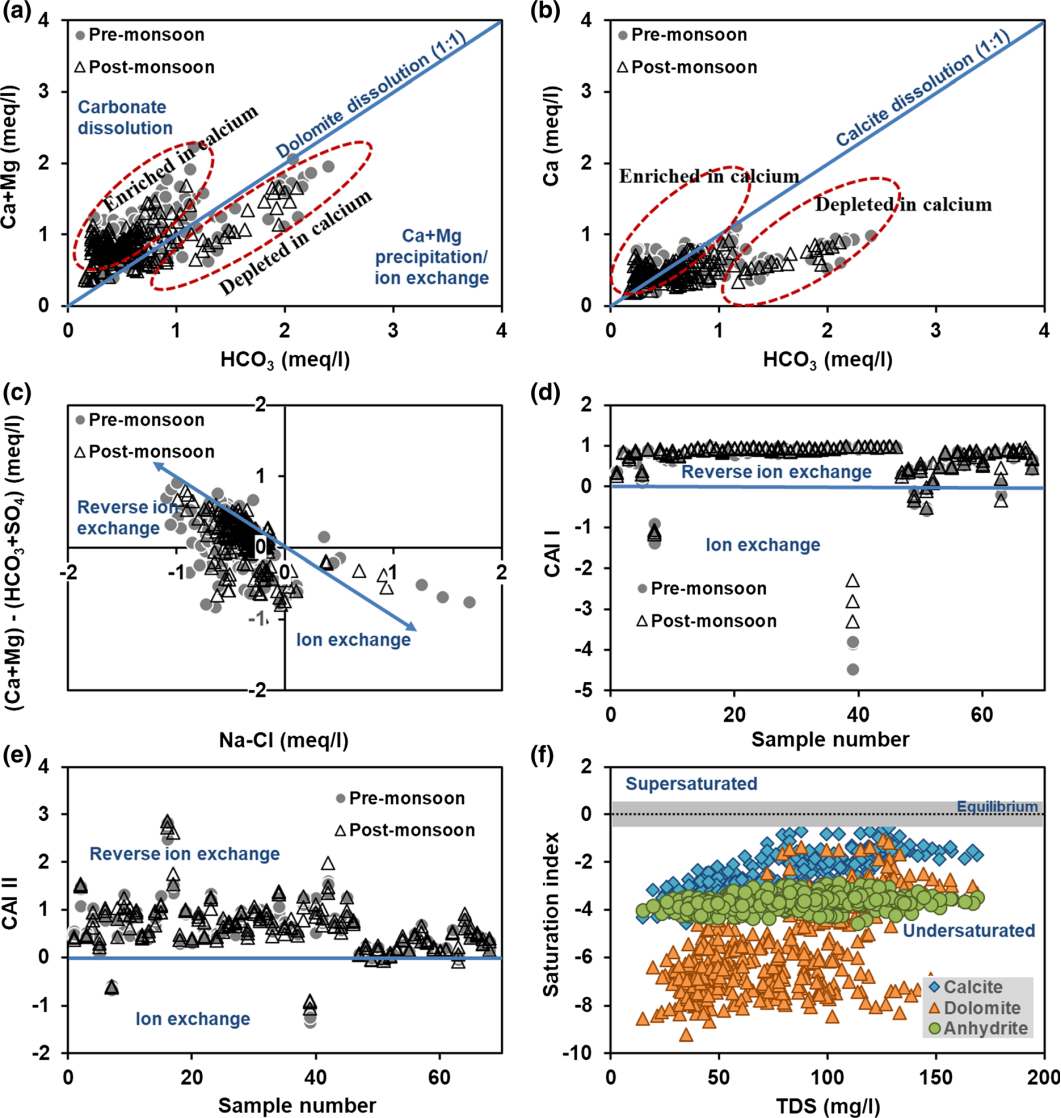
Cation exchange processes and saturation indices of selected minerals

If Ca^2+^, Mg^2+^ and HCO_3_^−^ are derived from dissolution of carbonate rocks, then Ca/HCO_3_ ratio will be 1:2, and (Ca + Mg)/HCO_3_ will be 1:1 in groundwater. Groundwater samples falling above the 1:1 equiline indicate carbonate dissolution (Fig. 4a). Samples below this line have deficit calcium and magnesium and can be explained by calcium and magnesium precipitation or cationic exchange of these ions against sodium, by weathering of silicate minerals (Bouzourra et al. 2015). An average (Ca + Mg)/HCO_3_ ratio of 1.72 and the (Ca + Mg)/total cations ratio of 0.89 suggest that silicate weathering in addition to dissolution of carbonate minerals governs the hydrogeochemical processes in the region.

#### Cation exchange process

The cation exchange by leaching or dissolution of carbonate and sulphate minerals can be differentiated. Groundwater samples distributed on and above the 1:1 line in Fig. 4a and b show an increase in Ca^2+^ from dolomite and calcite dissolution respectively. Samples distributed below this line have low calcium and are explained by precipitation of the carbonate minerals or weathering of silicate minerals through the cation exchange of calcium by sodium (Bouzourra et al. 2015). The direct ion exchange and reverse ion exchange can be differentiated through a plot Na–Cl vs Ca + Mg − HCO_3_ − SO_4_. If the relation between these two is linear with a slope of − 1, then the occurrence of reverse ion exchange is confirmed (Fig. 4c). With a slope of − 0.9 for premonsoon and − 0.8 for postmonsoon, reverse ion exchange is the dominant process; direct ion exchange is also noticed in few samples.

Chloroalkaline indices (CAI) I and II, calculated using Eq. 10 and 11, also suggest that reverse ion exchange dominates direct ion exchange (Fig. 4d, e).

10 $${\text{CAI I}} = {\text{Cl}} - \frac{{\left( {{\text{Na}} + {\text{K}}} \right)}}{\text{Cl}}$$

11 $${\text{CAI II}} = {\text{Cl}} - \frac{{\left( {{\text{Na}} + {\text{K}}} \right)}}{{{\text{SO}}_{4} + {\text{HCO}}_{3} + {\text{CO}}_{3} + {\text{NO}}_{3} }}$$

All values in meq/l. With exchange between Ca^2+^ or Mg^2+^ in groundwater with Na^+^ and K^+^ in the aquifer material, these indices are negative, indicating ion exchange. If there is exchange between Na^+^ or K^+^ in groundwater with Ca^2+^ or Mg^2+^ in the aquifer material, both the indices will be positive, indicating reverse ion exchange. CAI I ranged from − 4.48 to 0.99, and CAI II ranged from − 1.34 to 2.86.

### Geochemical modelling

The following parameters were used to calculate the SI of minerals using the geochemical model: pH, major cations, major anions and fluoride. All groundwater samples were undersaturated with carbonate minerals. SI of calcite varied from − 4.6 to − 0.6 and for dolomite the range was − 9.2 to − 1.1. Such highly undersaturated conditions of these minerals suggest the dissolution of the carbonate minerals. Normally, the SI moves closer to equilibrium with increase in TDS due to longer residence times (Fig. 4f).

### Trace element geochemistry

Distribution of trace metals in groundwater was in the following order: iron, arsenic, manganese, zinc, copper, lead, chromium and cadmium. Iron, manganese and zinc was recorded in all the locations (sampling locations = 68, total number of samples collected = 408) during the entire sampling period. Lead, copper and chromium was recorded in 170, 318 and 132 groundwater samples, respectively. Cadmium was found only in 3 locations and recorded 9 times. Arsenic was found only in 4 locations but existed consistently in these 4 locations throughout the study. Copper, zinc and chromium were within the standard limits in all the samples. Among the measured trace metals, iron exceeded the Bureau of Indian Standards (BIS) limits in 341 samples and lead exceeded in 38% of the samples (BIS 2012). Manganese was above prescribed limits in 7%, cadmium in 2% and arsenic in 0.5% of the groundwater samples.

Of the various trace metals studied, iron in groundwater poses a serious issue in the entire Tripura state. In north Tripura, iron up to 12 mg/l and in south Tripura up to 3.7 mg/l have been reported (CGWB 2012a, b). The Tipam Sandstone aquifers of the region are ferruginous in nature. Iron concentration was generally found to be lower in open wells than the tube wells in the northern and southern parts of Tripura as the open wells facilitate aeration allowing the precipitation of ferrous iron as ferric iron (CGWB 2012a, b). However, high iron content in relation to open-tube borehole could not be identified in this study, as only 2 sampling wells were from shallow open wells and the others were tube wells. Excessive iron, arsenic and other trace metals in groundwater is non-potable and carries health risk if consumed. The adverse effects of exposure to high concentration of iron over prolonged period include gastrointestinal irritation, nausea and vomiting (USEPA 2006). Arsenic has carcinogenic properties and is known to cause dermal effects such as skin lesions, cardiovascular, respiratory, gastrointestinal, reproductive, developments effects and can be lethal at high doses (Chakraborti et al. 2015, 2017b; Rahaman et al. 2013). Hence, the concentration of these trace metals should be lowered to the desirable limit before using for domestic purposes.

Usually, the solubility of trace metals is low and hence they are measured in low concentrations in groundwater. But in acidic groundwater (low pH), the solubility and mobility of trace metals are increased. Variation in the redox conditions in groundwater strongly influences its trace metals concentration. Oxidation of organic matter present in the sub-surface enhances the redox processes, and the local hydrogeology and long residence times influence the migration of trace metals in groundwater. The reducing conditions also prove favourable for microbes to enable the transfer of electrons between different ions (Jahanshahi and Zare 2015; McMahon and Chapelle 2008; Palmucci et al. 2016). Redox potential was not measured during the field visit, which is a limitation of this study. Use of agrochemicals for agriculture may also have contributed to trace metals in groundwater to some extent. But any other contribution from anthropogenic sources such as industries or mining can be safely overlooked as such activities do not occur in the study area.

**Fig. 5:**
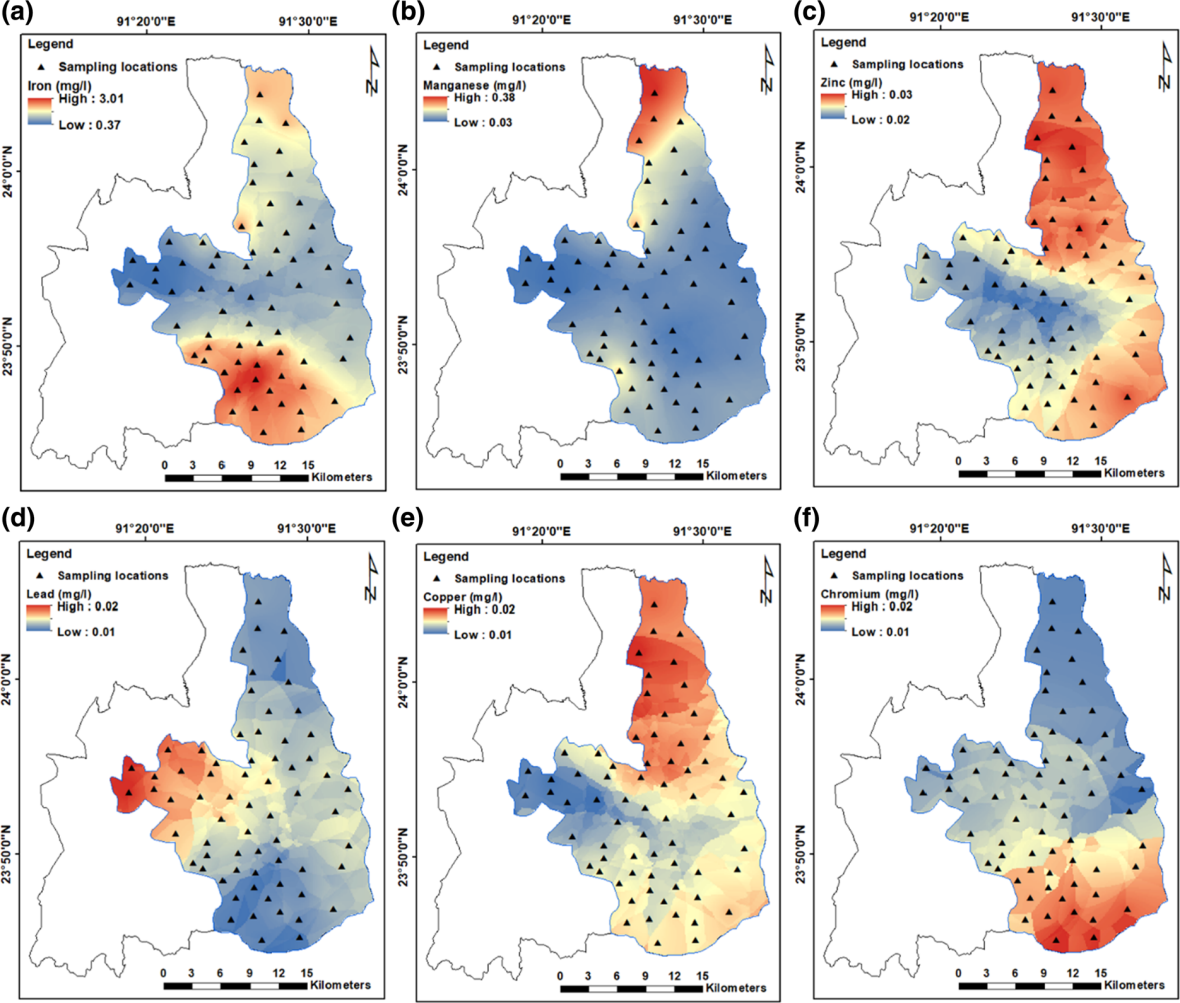
Spatial distribution in the concentration of various trace metals (based on the average concentration measured in each sampling location) using the empirical Bayesian kriging method

Geostatistical modelling using EBK was performed to spatially interpolate the concentration of trace metals measured in the study. Spatial variation in the average concentration of trace metals based on the EBK interpolation is given in Fig. 5a–f. Concentration of most trace metals is higher on the northern parts of the study area. The spectrum of semivariogram models for selected parameters is shown in Figure S1a-f (Supplementary material). The red solid line indicates the median of distribution, and the 25th and 75th percentiles are depicted with red dashed lines. The blue lines indicate each semivariogram model, and the thickness of the blue line is directly proportional to the semivariogram weights, i.e. models with smaller weights are shown as thin blue lines and models with higher weights are shown as thicker blue lines (Krivoruchko 2012). Blue crosses represent the empirical semivariances. For a valid model, the root-mean-square and the average standard errors are smaller with the root- mean-square standardized being close to one (Krivoruchko 2011). The root-mean-square standardized values in this study were close to one, symbolizing a valid prediction (Table S2). Similarly, the average standard errors are also small (value) indicating a true model. A summary of the predicted error statistics for selected parameters is given in Table S2, and comparison of the simulated and observed values is shown in Figure S3. Geostatistical analysis could not be performed for arsenic and cadmium due to insufficient data, i.e. the number of samples with recorded concentration of these trace metals was < 5.

### Anthropogenic contamination

**Fig. 6:**
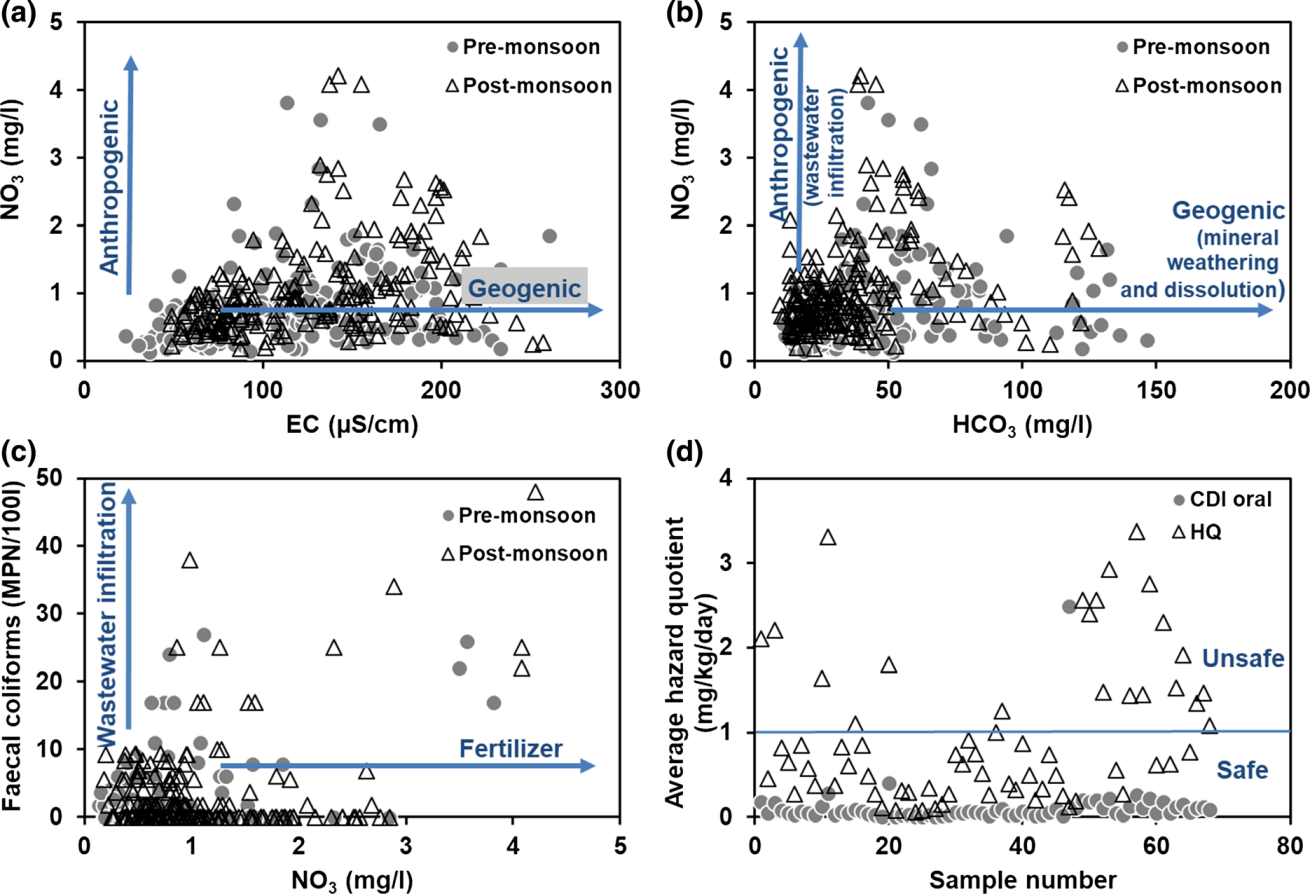
Plots depicting anthropogenic sources of pollution and human health risk based on hazard quotient

Hydrochemistry in the region is affected by anthropogenic sources like contamination from wastewater leakage from sewage tanks and agricultural fertilizers. During field survey among the tribal people of the region, it was noted that many suffer from diarrhoea mostly during the rainy season. Distance between the toilets and hand pumps are closely located, and they do not have proper sanitary seal which has a greater chance for bacterial contamination of groundwater. In this area, shallow tube wells are drilled manually and not well-constructed. Total coliforms were determined in 37% of groundwater samples. A total of 31% and 43% of samples had bacterial contamination in pre- and postmonsoon, respectively. Faecal coliforms were present overall in 32% of the groundwater samples, while 25% of the samples from premonsoon and 39% of the samples from postmonsoon were contaminated. Nitrate concentration in groundwater was at low concentrations with a maximum recorded value of 4.2 mg/l. Nitrate resulting from geogenic processes can be identified through bivariate plots of nitrate with EC and bicarbonate (Fig. 6a, b). This shows that nitrate is mostly contributed by anthropogenic sources. This also holds true for faecal coliforms as they showed a positive relationship with nitrate, indicating contamination from wastewater infiltration (Fig. 6c).

### Water quality index

**Fig. 7:**
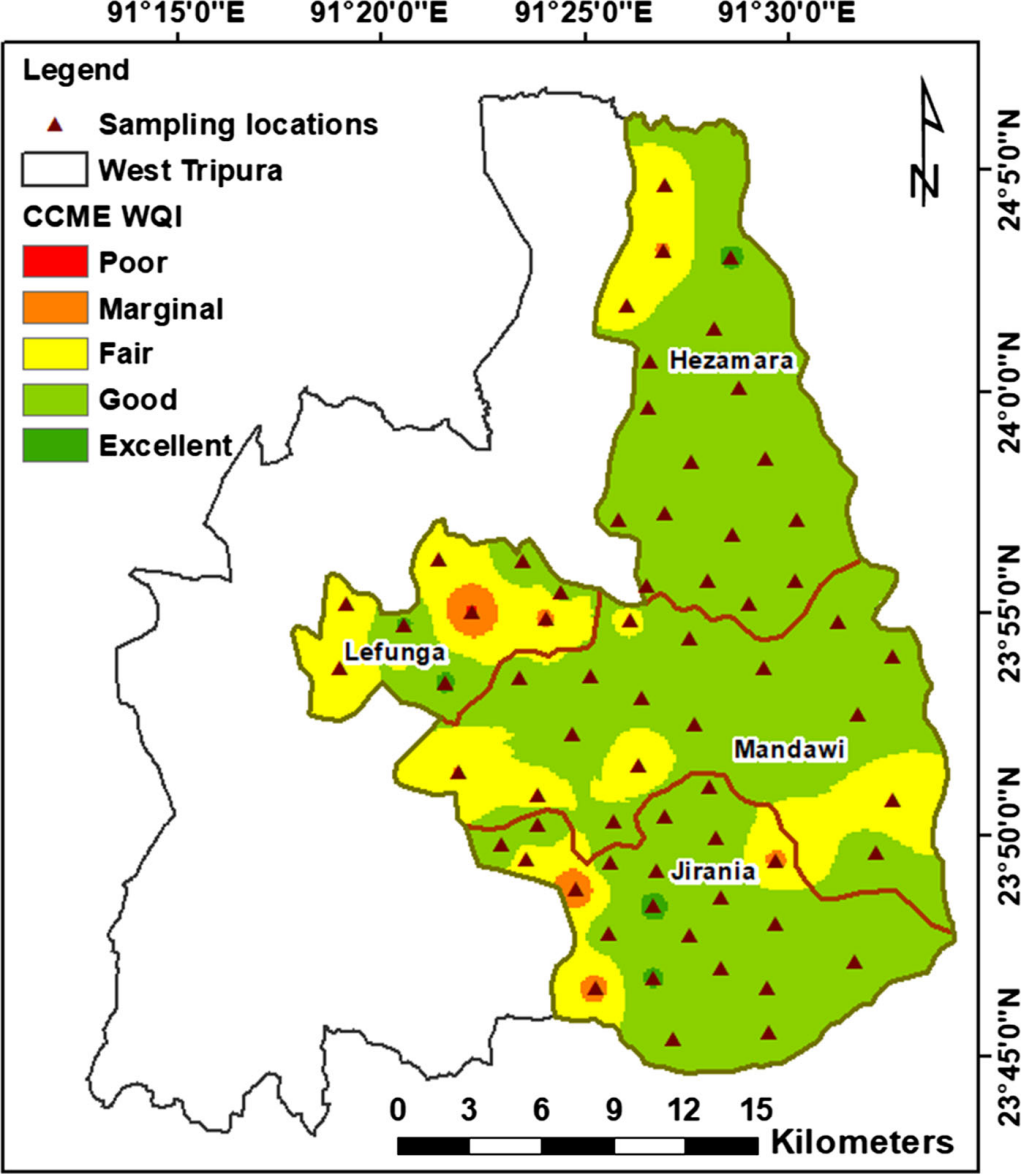
Groundwater quality index indicating the suitable and unsuitable areas

CCME WQI was calculated based on the following water quality parameters: pH, TDS, major cations and anions, nitrate, fluoride, trace metals and coliforms. The calculated CCME WQI varied from 42 to 100. They were classified as suggested by the CCME (2001) into five classes: excellent (95–100), good (80–94), fair (65–79), marginal (45–64) and poor (0–44). This classification is subjective and was put forth based on the information at hand, expert judgement and public’s expectations (CCME 2001). The WQI shows 1.5% had poor, 8.7% had marginal, 16.2% had fair, 66.2% had good, and 7.4% had excellent water quality. Spatial distribution of the CCME WQI (Fig. 7) shows that most of the area has good groundwater quality, i.e. within the prescribed limits of BIS (2012). Marginal groundwater quality is found at few locations in the western part of the study area. These locations are not grouped together, and the source of the pollution in these sampling locations should be studied individually in detail.

### Multivariate statistical analysis

Statistical analysis with all the measured parameters over the entire study did not show clear relationship among them as certain trace metals in several groundwater samples were below detection limit (BDL). Hence, factor analysis was performed for EC, major cations, major anions and selected trace metals. Parameters with more than 20% of the samples having concentrations BDL are eliminated from the analysis because including these parameters introduces uncertainties in the multivariate results. Initially, factor analysis extracted 13 components. Of these, only the first 5 components had eigenvalues  > 1 and account for a total cumulative variance of 64% (Table 2). Factor 1 has strong positive loadings in sodium and potassium indicating geogenic sources. Since in Factor 1 no other ions exhibit strong loadings, this could also be due to ion exchange process. Factor 2 has high positive loading for calcium, magnesium and bicarbonate and corresponds to calcite and dolomite weathering. Bicarbonate and nitrate showing positive loadings in Factor 3 indicate contribution from wastewater infiltration. Factor 4 have positive loadings for fluoride, iron and manganese and can be attributed to geogenic process such as weathering and redox reactions. Earlier studies have also reported that the mobility of iron and manganese is independent from the other major ions and shows negative correlation with nitrate (Palmucci et al. 2016; Paul et al. 2019a), similar to the present study. Hence, Factor 4 may be chiefly regulated by redox processes. Factors 5 has positive values for magnesium and zinc and may be attributed to anthropogenic sources such as fertilizer application.

**Table 2:** Factor loadings of the various parameters from the principle component extraction method

Parameter	Component				
	Factor 1	Factor 2	Factor 3	Factor 4	Factor 5
EC	0.1	− 0.4	− 0.5	0.0	− 0.3
Ca	0.1	**0.9**	0.0	0.0	0.0
Mg	0.2	**0.8**	0.1	0.2	− 0.1
Na	**0.8**	0.2	0.2	0.1	0.1
K	**0.8**	0.1	0.0	0.1	0.2
HCO_3_	− 0.4	**0.7**	**0.6**	0.1	0.1
Cl	− 0.3	0.3	0.0	− 0.3	0.5
SO_4_	0.2	0.2	0.7	0.2	− 0.1
NO_3_	0.3	− 0.1	**0.8**	− 0.1	− 0.1
F	0.0	0.1	0.1	**0.7**	− 0.1
Fe	0.1	0.1	0.0	**0.8**	**0.1**
Mn	0.2	0.0	0.0	**0.4**	**0.6**
Zn	0.1	− 0.1	0.0	0.0	**0.8**
Eigenvalues	2.8	1.7	1.4	1.3	1.1
Variance (%)	21.7	13.4	10.7	9.7	8.6
Cumulative variance (%)	21.7	35.1	45.9	55.6	64.2

Strong correlation is indicated by bold values

Correlation between the trace metals is not rather clear as all the samples do not have constant content of these ions compared to other parameters in the dataset and many samples had BDL values. Positive correlation between calcium, magnesium and bicarbonate confirms the carbonate minerals weathering and dissolution (Table 3). Positive correlation between iron and manganese was also noticed in part of the study areas earlier by indicating reduction of iron hydroxides and manganese oxides (Paul et al. 2019a). The results from factor analysis and correlation studies are consistent with the sources and hydrogeochemical processes identified. Detailed studies on trace metals in groundwater are required.

**Table 3:** Correlation among various groundwater parameters

	EC	Ca	Mg	Na	K	HCO_3_	Cl	SO4	NO_3_	F	Fe	Mn	Zn
EC	1												
Ca	0.50	1											
Mg	0.47	0.81	1										
Na	0.18	0.02	0.11	1									
K	0.13	0.02	0.06	0.96	1								
HCO_3_	0.57	0.57	0.66	0.27	0.20	1							
Cl	− 0.01	0.21	0.16	− 0.11	− 0.09	− 0.11	1						
SO_4_	0.20	0.32	0.37	0.49	0.45	0.22	− 0.04	1					
NO_3_	0.32	0.08	0.11	0.56	0.54	0.21	− 0.05	0.52	1				
F	0.01	0.06	0.22	0.21	0.23	0.17	− 0.06	0.33	0.12	1			
Fe	0.20	0.18	0.28	0.09	0.07	0.35	− 0.11	0.11	0.06	0.40	1		
Mn	− 0.02	0.02	0.05	0.11	0.13	0.00	0.07	0.02	− 0.02	0.28	0.51	1	
Zn	− 0.04	0.01	− 0.01	0.02	− 0.02	0.02	0.15	0.09	− 0.03	− 0.03	0.16	0.27	1

### Human exposure risk assessment

All major and minor ions did not pose a major threat to human health when compared with the BIS standards (Table 1). Hence, the human health risk was calculated only for the trace metals. Input for calculating the risk and the statistical summary of the human health risk from the trace metals are given in Tables 4 and 5, respectively. Both CDI and HQ within 1 mg/kg/day are safe and above this value are harmful to human health. CDI_oral_ for the trace metals was in the following order: iron > manganese > zinc > copper > lead > chromium > arsenic > cadmium. HQ (non-carcinogenic risk) ranged from 0 to 3.8 mg/kg/day (Fig. 6d), and the HQ of individual trace metals was in the order of iron > zinc > manganese > copper > lead > chromium > arsenic > cadmium (Table 5). CDI_oral_ of individual trace metals did not exceed 1 mg/kg/day. HQ of individual metals was within safe limit for all trace metals except for iron. HQ for iron ranged from 0.04 to 3.77 mg/kg/day, and 32% of the samples had HQ > 1 mg/kg/day. Long-term exposure to arsenic and iron through the oral pathway can cause many serious health problems. This area has unsafe levels of iron and arsenic in groundwater, and the risk to human health cannot be ignored.

**Table 4:** Input data for calculation of human exposure risk through the drinking water pathway

Parameter for oral ingestion (unit)	Values		Reference
C = measured concentration of the trace metal in water (mg/l)	Measured values		–
IR = average daily water intake (l/day)	3		Planning commission (2011)
EF = exposure frequency (days/year)	365		–
ED = exposure duration (years)	66.4		UNDESA (2013)
BW = average body weight (kg)	57.5		ICMR (2009)
AT = average life expectancy (days)	365 X 66.4 = 24,236		–
RfD = oral reference dose for a trace metal that an individual can be exposed to in a day over his/her lifetime without experiencing any harmful health effect (mg/kg-day)	Fe	7.0E−01	USEPA (2006)
	Mn	5.0E−03	IRIS (undated-b)
	Pb	3.6E−03	Viridor Waste Ltd (2009)
	Cd	5.0E−04	IRIS from USEPA (2009)
	As	3.0E−04	IRIS (undated-a)
	Cu	5.0E−03	USEPA from CHMP (2007)
	Zn	3.0E−01	IRIS (2005)
	Cr	3.0E−03	IRIS from USEPA (2009)

**Table 5:** Human health risk associated with groundwater used for drinking

Human exposure risk	Trace metal	Number of samples where trace metal concentration was above BDL	Min	Max	Mean	Sum	% exceeding 1 mg/kg/day
Chronic daily intake (mg/kg/day)	Iron	408	2.6E−03	2.8E−01	7.1E−02	2.9E+01	Nil
	Manganese	408	2.6E−04	3.2E−02	5.1E−03	2.1E+00	Nil
	Lead	170	5.2E−04	1.8E−03	7.9E−04	1.3E−01	Nil
	Cadmium	9	2.6E−04	4.2E−04	3.0E−04	2.7E−03	Nil
	Arsenic	24	5.6E−05	2.7E−03	7.2E−04	1.7E−02	Nil
	Copper	318	1.0E−04	2.0E−03	7.6E−04	2.4E−01	Nil
	Zinc	408	4.7E−04	5.4E−03	1.2E−03	4.8E−01	Nil
	Chromium	132	5.2E−04	1.6E−03	7.6E−04	1.0E−01	Nil
	Total	408	3.8E−03	3.1E−01	7.8E−02	3.2E+01	Nil
Hazard quotient (mg/kg/day)	Iron	408	3.5E−02	3.8E+00	9.5E−01	3.9E+02	32
	Manganese	408	2.5E−05	3.1E−03	4.9E−04	2.0E−01	Nil
	Lead	170	3.6E−05	1.2E−04	5.4E−05	9.2E−03	Nil
	Cadmium	9	2.5E−06	4.0E−06	2.8E−06	2.6E−05	Nil
	Arsenic	24	3.2E−07	1.5E−05	4.2E−06	1.0E−04	Nil
	Copper	318	1.0E−05	2.0E−04	7.3E−05	2.3E−02	Nil
	Zinc	408	2.7E−03	3.1E−02	6.7E−03	2.8E+00	Nil
	Chromium	132	3.0E−05	9.3E−05	4.4E−05	5.7E−03	Nil
	Total	408	3.8E−02	3.8E+00	9.6E−01	3.9E+02	32

## Conclusion

Hydrogeochemical and geostatistical methods were successfully applied to evaluate the trace metal contamination in groundwater in a part of West Tripura, north-eastern India. Influence of natural recharge on shallow groundwater levels during postmonsoon and the decrease in water table due to local pumping activities for domestic purpose and agriculture use were witnessed. Carbonate dissolution, silicate weathering and cation exchange were the key geochemical processes responsible for groundwater mineralization. Copper, zinc and chromium were within the prescribed limits. Iron, manganese, lead, cadmium and arsenic were the above limits in 84%, 7%, 38%, 2% and 0.5% of the samples. Mobilization of most of these trace metals is governed by oxidizing and reducing conditions. Contamination from faecal coliforms was apparent after monsoons affecting human health. The results from factor analysis and correlation studies are consistent with the sources and hydrogeochemical processes identified. Groundwater is not potable in the region, and alternate source of freshwater for domestic needs is essential.

## Electronic supplementary material

Below is the link to the electronic supplementary material.

Supplementary material 1 (DOCX 3009kb)
