# Supplementary material for: Trace metals contamination in groundwater and implications on human health: comprehensive assessment using hydrogeochemical and geostatistical methods
Source: Environ Geochem Health. 2020 Jun 29;42(11):3819–39. doi: 10.1007/s10653-020-00637-9 (PMC7641953; doi:10.1007/s10653-020-00637-9)

Supplementary material

**Trace metals contamination in groundwater and implications on human health: comprehensive assessment using hydrogeochemical and geostatistical methods**

K. Brindha, Rajib Paul, Julien Walter, Mou Leong Tan and Mahesh Kumar Singh

Environ Geochem Health

https://doi.org/10.1007/s10653-020-00637-9

| **Table S1 Method of analysis and detection limit of various parameters** | | |
| --- | --- | --- |
| **Parameter** | **Method of analysis** | **Minimum detectable limit** |
| pH | pH meter (Eutech Instruments pH-700) | - |
| Conductivity | Conductivity meter (Metler Toledo AG,6803) | 5 µS/cm |
| TDS | Gravimetric Method | 5 mg/l |
| Ca | EDTA, Titrimetric Method | - |
| Mg | EDTA, Titrimetric Method | - |
| Na | Atomic Absorption Spectrometer (Perkin Elmer A Analyst 700). | 0.001 mg/l |
| K | Atomic Absorption Spectrometer (Perkin Elmer A Analyst 700). | 0.001 mg/l |
| HCO3 | Titration Method | 5 mg/l |
| Cl- | Argentometric Titration Method | 1 mg/l |
| SO4-2 | Turbidimetric Method | 1 mg/l |
| NO3- | UV-Spectrophotometric method | 0.1 mg/l |
| F- | SPADNS Method | 0.1 mg/l |
| Fe | Atomic Absorption Spectrometer (Perkin Elmer A Analyst 700). | 0.001 mg/l |
| TH | EDTA, Titrimetric Method | 1 mg/l |
| Mn | Atomic Absorption Spectrometer (Perkin Elmer A Analyst 700). | 0.001 mg/l |
| Pb | Atomic Absorption Spectrometer (Perkin Elmer A Analyst 700). | 0.003 mg/l |
| Cd | Atomic Absorption Spectrometer (Perkin Elmer A Analyst 700). | 0.003 mg/l |
| As | Atomic Absorption Spectrometer (Perkin Elmer A Analyst 700) with Mercury Hydride Generation System (MHS-15) | 0.2 µg/l |
| Cu | Atomic Absorption Spectrometer (Perkin Elmer A Analyst 700). | 0.001 mg/l |
| Zn | Atomic Absorption Spectrometer (Perkin Elmer A Analyst 700). | 0.001 mg/l |
| Cr | Atomic Absorption Spectrometer (Perkin Elmer A Analyst 700). | 0.001 mg/l |
| TC | Multiple tube fermentation technique (MPN test) | - |
| FC | Multiple tube fermentation technique (MPN test) | - |

| Table S2 Statistical summary of prediction error from empirical Bayesian kriging | | | | | | |
| --- | --- | --- | --- | --- | --- | --- |
| **Trace metal** | **N** | **Mean** | **Root-mean-square (RMS)** | **Standardised mean** | **Standardised RMS** | **Average standard error** |
| Iron | 68 | 6.8E-03 | 1.1E+00 | 6.3E-03 | 9.7E-01 | 1.2E+00 |
| Manganese | 68 | 1.4E-03 | 9.4E-02 | 9.4E-03 | 1.0E+00 | 9.4E-02 |
| Zinc | 68 | 4.4E-04 | 8.1E-03 | 5.3E-02 | 1.0E+00 | 8.0E-03 |
| Lead | 43 | 1.8E-04 | 3.5E-03 | 4.7E-02 | 9.7E-01 | 3.6E-03 |
| Copper | 65 | 2.3E-04 | 5.2E-03 | 4.2E-02 | 9.9E-01 | 5.2E-03 |
| Chromium | 34 | 4.4E-06 | 4.3E-03 | 2.3E-04 | 1.0E+00 | 4.4E-03 |
| Arsenic | 4 | Unable to compute due to insufficient data | | | | |
| Cadmium | 3 | Unable to compute due to insufficient data | | | | |


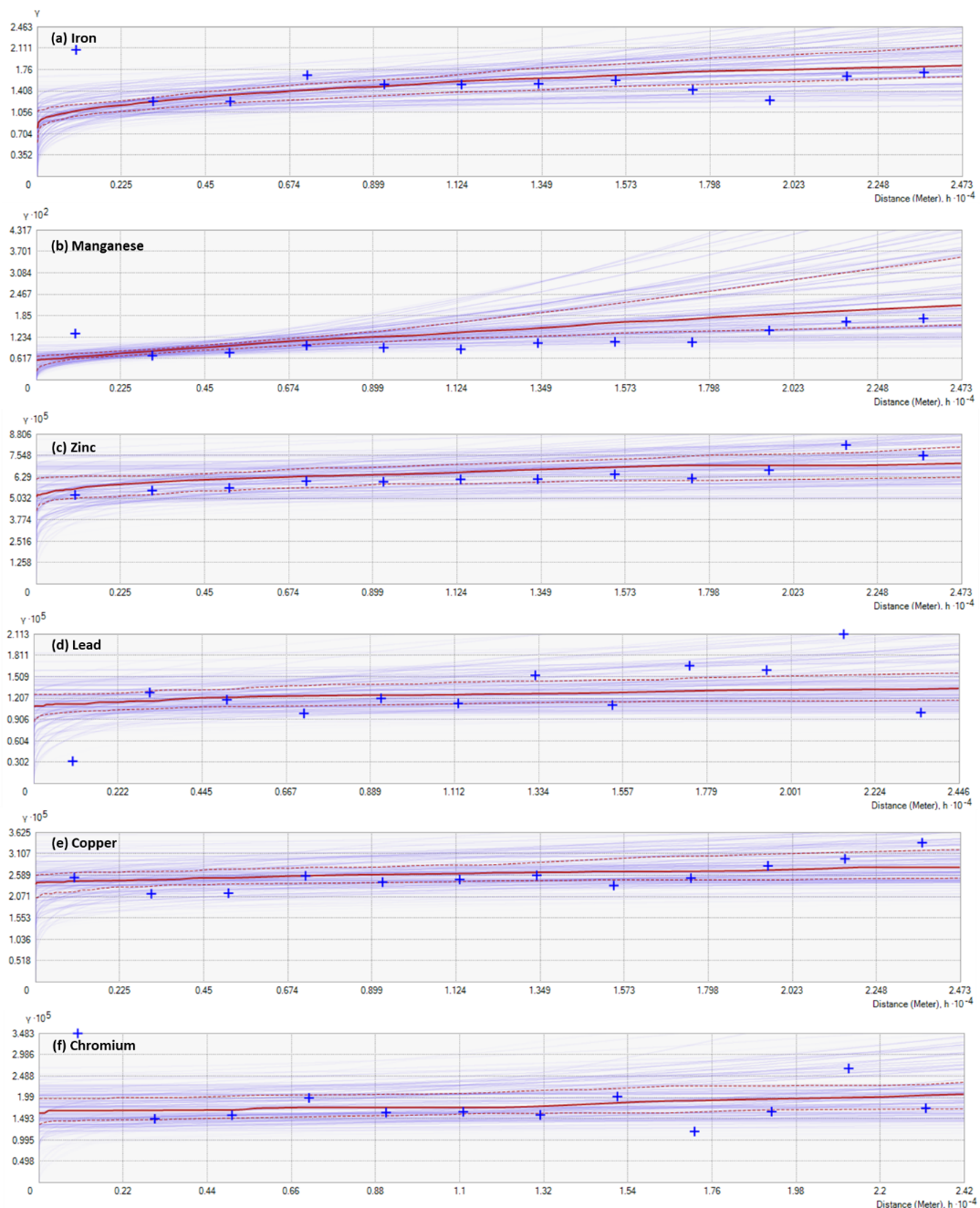


**Figure S1 Semivariograms of the trace metals using the empirical Bayesian kriging method**

**Figure S2 Predicted versus measured trace metal concentrations in groundwater derived from empirical Bayesian kriging**


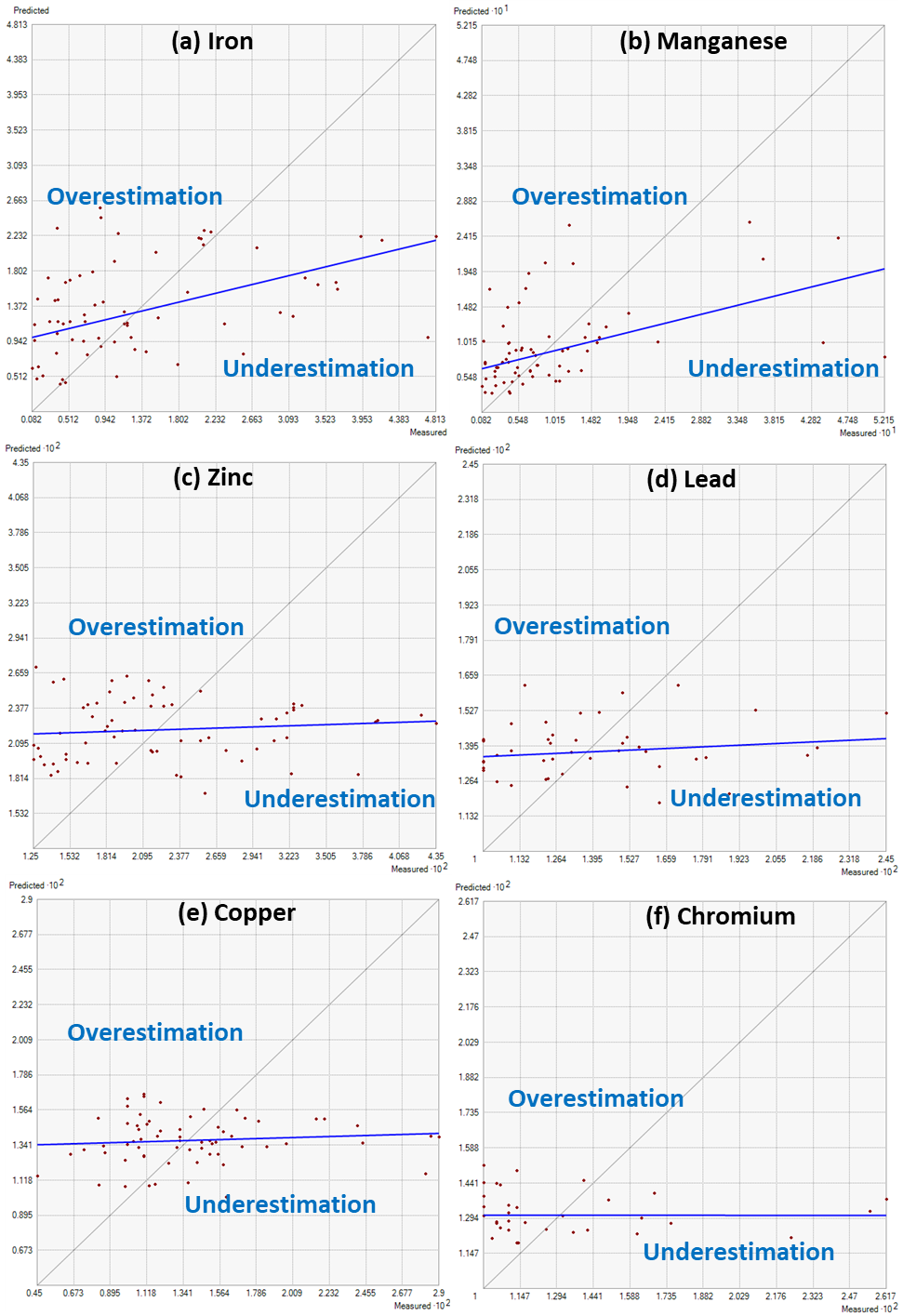

Supplement: Supplementary file 1 — Supplementary material 1 (DOCX 3009kb) [file 10653_2020_637_MOESM1_ESM.docx]
